# Sex influences the association between haemostasis and the extent of lung lesions in tuberculosis

**DOI:** 10.1186/s13293-018-0203-9

**Published:** 2018-10-10

**Authors:** Wenling Tan, Adiilah K Soodeen-Lalloo, Yue Chu, Weijie Xu, Fengfang Chen, Jie Zhang, Wei Sha, Jin Huang, Guanghong Yang, Lianhua Qin, Jie Wang, Xiaochen Huang, Jingyun Shi, Yonghong Feng

**Affiliations:** 1 Shanghai Key Laboratory of Tuberculosis, Shanghai Pulmonary Hospital, Tongji University School of Medicine, 507 Zhengmin Road, Shanghai, 200433 China; 2 Department of Radiology, Shanghai Pulmonary Hospital, Tongji University School of Medicine, Shanghai, 200433 China; 3 Department of Clinical Laboratory Medicine, Shanghai Pulmonary Hospital, Tongji University School of Medicine, Shanghai, 200433 China; 4 Key Laboratory of Environment Pollution Monitoring and Disease Control, Ministry of Education, School of Public Health, Guizhou Medical University, Guiyang, 550025 Guizhou China; 5 Department of Epidemiology and Biostatistics, Tongji University School of Medicine, Shanghai, 200433 China; 6 Clinic and Research Centre of Tuberculosis, Shanghai Pulmonary Hospital, Tongji University School of Medicine, Shanghai, 200433 China

**Keywords:** Tuberculosis, Gender bias, Coagulation, Platelet

## Abstract

**Background:**

Worldwide tuberculosis (TB) reports show a male bias in morbidity; however, the differences in pathogenesis between men and women with TB, as well as the mechanisms associated with such differences, are poorly investigated. We hypothesized that comparison of the degree of lung injury and clinical indices of well-matched men and women with newly diagnosed TB, and statistical analysis of the correlation between these indices and the extent of lung lesions, can provide insights into the mechanism of gender bias in TB.

**Methods:**

We evaluated the acid-fast bacilli grading of sputum samples and compiled computed tomography (CT) data of the age-matched, newly diagnosed male and female TB patients without history of smoking or comorbidities. Inflammatory biomarker levels and routine haematological and coagulation-associated parameters were compared. Binary logistic regression analysis was used to define the association between the indices and lung lesions, and the influence of sex adjustment.

**Results:**

Women with TB have a longer delay in seeking healthcare than men after onset of the TB-associated symptoms. Men with TB have significantly more severe lung lesions (cavities and healing-associated features) and higher bacterial counts compared to women with TB. Scoring of the CT images before and after anti-TB treatment showed a faster response to therapy in women than in men. Coagulation- and platelet-associated indices were in models from multivariate regression analysis with groups of males or females with TB or in combination. In univariate regression analysis, lower lymphocyte counts were associated with both cavity and more bacterial counts, independent of sex, age and BMI. The association of international normalized ratios (INR), prothrombin times (PTs), mean platelet volumes (MPVs) and fibrinogen (FIB) level with lung lesions was mostly influenced by sex adjustment.

**Conclusions:**

Sex influences the association between haemostasis and extent of TB lung lesions, which may be one mechanism involved in sex bias in TB pathogenesis.

**Electronic supplementary material:**

The online version of this article (10.1186/s13293-018-0203-9) contains supplementary material, which is available to authorized users.

## Background

It is estimated that one third of the world’s population is infected with *Mycobacterium tuberculosis* (*Mtb*.), the pathogen that causes tuberculosis (TB). TB was directly responsible for approximately 1.7 million deaths in 2016 and remains one of the top 10 causes of death worldwide despite the availability of drug treatment [1]. Epidemiological surveys have indicated that men tend to be affected more than women, with a man to woman ratio close to 2 as per the worldwide case notification rates [1]. The degree of male bias varies by geographic location and by year, but the overall trend is clear [2].

While disagreement exists on whether socioeconomic and cultural factors may create barriers to accessing healthcare which hence cause undernotification in women, particularly in developing countries [3–5], in recent years, however, increasing numbers of studies have indicated that biological mechanisms may actually account for a significant part of the difference between men and women in susceptibility to infection, including TB [6–8]. Consistently, with C57BL/6 and Balb/c mouse models, previous researches have demonstrated that infection with *Mtb*. resulted in exacerbated pulmonary pathology and increased morbidity and mortality in male mice compared to females [9, 10]. The study showed that female and castrated male mice exhibited significant higher inflammation (higher TNF-α, IFN γ, IL12, iNOS and IL17) in all lung compartments than non-castrated males during the first month of infection [10]. In a genotyping study based on a South African population, Salie et al. found sex-specific associations for TLR8 polymorphisms with susceptibility to TB [11]. Nevertheless, evidence for the sex bias in TB pathogenesis based on clinical observation is still scarce, and the underlying mechanisms are still little known.

In this study, we compared the bacterial counts in sputum and the severity of lung lesions on computed tomography (CT) between two groups of age-matched men and women with newly diagnosed TB. We hypothesize that comparison of the biochemical and immunological indices between the groups followed by regression analyses, with and without adjustment for sex, will reveal the indices involved in sex bias in TB-associated lung lesions.

## Methods

### Study subjects

We included primary pulmonary TB patients from Shanghai Pulmonary Hospital (SPH) between April 2011 and April 2015. TB was diagnosed based on acid-fast bacilli (AFB) staining and culture; patients whose cultures yielded nontuberculous mycobacteria were excluded from the study. We retrospectively reviewed medical records and excluded patients who had history of any of the following: smoking, excessive alcohol drinking, human immunodeficiency virus (HIV) infection, immunosuppressive drug therapy, hormone therapy, cancer, diabetes, pneumoconiosis, silicosis and hepatitis B and C viruses’ infection. In the enrolled 114 pairs of male and female patients matched for age (± 3 years), none of the patients had received or initiated anti-TB drug therapy for more than 1 week before registration at SPH.

Documented data (complete blood count, CBC) from 62 pairs of healthy men (30.2 ± 6.7 years) and women (29.8 ± 6.9 years) employees in SPH who had health examination at the same period were used as controls.

### Review of clinical findings and laboratory tests

We retrospectively reviewed patient characteristics including age, sex, height, weight, body mass index (BMI), symptoms, cavity and sputum smear grade. Changes in inflammatory biomarker levels and routine haematological and biochemical parameters were also reviewed. All data were obtained from the first medical records during patient admission.

### T-SPOT.TB tests and anti-TB IgG antibody detection

T-SPOT.TB and serum anti-TB IgG antibody detection were carried out using commercially available T-SPOT.TB kits (Oxford Immunotec. Ltd) and ELISA kits based on a dot immunogold filtration assay (DIGFA) with specific membrane antigens from *Mtb*. bound to a nitrocellulose filter membrane (Shanghai Aopu Biomed Ltd. Company, China), respectively. Blood samples were collected and peripheral blood mononuclear cells (PBMCs) were prepared within 2 h as per standard protocol. All procedures, including interpretation of the results, were carried out according to the manufacturer’s recommendations. The absolute numbers of spot-forming cells (SFCs) were counted using an Elispot counter (AID, Strasberg, Germany), and the numbers of antigen-specific SFCs in positive T-SPOT.TB assays were plotted following the subtraction of the numbers in the negative control.

### AFB grading

The Auramine O fluorescent staining method was used to stain the sputa [12]. The grades of the AFB smear-positive samples were reported according to the Standard Procedures for Laboratory Diagnosis of Tuberculosis by the Chinese Anti-Tuberculosis Association as follows [13]: grade 1 (1+), 10–99 bacilli/50 fields; grade 2 (2+), 1–9 bacilli/field; grade 3 (3+), 10–99 bacilli/field; and grade 4 (4+), 100 bacilli/field. The highest grade for each individual within the first week of admittance was selected for analysis. Patients were divided into two groups with low (1+ and 2+) and high (3+ and 4+) sputum bacteria counts for binary regression analysis.

### Computed tomography evaluation

In total, high-resolution computed tomography (HRCT) scans from 94 female and 95 male patients performed within the first week of admittance (T0) were collected. The scans were assessed by two specialists who were blinded to the groups of patients. Lungs were divided into six zones (low, middle and high zones for the left and right lungs), and the presence of abnormalities including nodule, micronodule, cavity, consolidation, parenchymal bands, ground glass opacity and bronchial lesion was noted according to previous reports [14, 15]. The total weighted HRCT score was calculated as HRCT score × 100/168 (total score) + 40 if cavitation was present.

Descriptive terms used to interpret the CT findings were defined as previously reported [16]. Cavity wall thickness > 3 mm was defined as thick-walled, and cavity wall thickness ≤ 3 mm was defined as thin-walled.

### Follow-up analysis of the response to anti-TB treatment

The responses of the patients to standard anti-TB treatment (a standard regimen consisting of isoniazid, rifampin, pyrazinamide and ethambutol (2HRZE/4HRE) according to the National TB Programme) were evaluated by tracking the records of sputum bacterial grades and CT imaging after 1 (T1) and 3 months (T3) of therapy.

In total, records of sputum bacterial counts from 87 females and 79 males were tracked and scored. Thirty-four female and 24 male patients at T1 and 36 female and 28 male patients at T3 had CT scan records; only 15 female and 7 male patients had complete records of CT scans at T0, T1 and T3.

### Ethical approval

This study was conducted in accordance with the amended Declaration of Helsinki and the ethical guidelines of the institutional review board of Tongji University (project approval number 2014fk10). All participants gave written consent for the use of their clinical information for research purposes. Clinical data were anonymized.

### Statistical analyses

We performed *χ*^2^ test for categorical variables, Wilcoxon rank-sum test for nominal variables and *t* tests for continuous variables. To identify the parameters associated with the extent of lung lesions (sputum bacterial counts and cavity), 114 pairs of male and female TB patients with complete records of 48 physiological, haematological and biochemical analyses were chosen for multivariate (male and female groups separately and in combination) and univariate (combination of male and female cases) logistic regression analyses. In univariate regression analysis, the association was adjusted for age/BMI and sex, separately or in combination, to test the influence of these factors on the association. Statistical significance was determined at *P* < 0.05. All analyses were performed using SPSS (version 19, SPSS Inc., Chicago, IL, USA).

## Results

### Patient characteristics

Although the 114 pairs of age-matched female (35.2 ± 14.0 years) and male (35.1 ± 13.9 years) TB patients had significant differences in height and weight, they showed no significant differences in BMI at the time of first registration (F 19.3 ± 2.4 versus M 19.8 ± 2.6; *P* = 0.136). Besides, ratios of cases with BMI < 18.5 (malnutrition) were without statistical difference in the two groups (F 38.5% versus M 31.4%, *P* = 0.276). Intriguingly, a significant difference was found between the groups in time elapsed between onset of symptoms and seeking medical care. A higher ratio of men with TB, compared to women with TB, sought healthcare within 1 month of onset of symptoms (32.5% versus 14.9%); more than half of the female patients (59.6%) sought healthcare between 1 and 6 months after onset of symptoms (Table 1).

**Table 1:** Characteristics of study patients

	Sputum smear-positive TB		*P* value
	Female (*n* = 114)	Male (*n* = 114)	
Age, mean (sd), year	35.2 (14.0)	35.1 (13.9)	0.943^a^
Height, cm^†^	160 (158–164) *n* = 109	173 (170–177) *n* = 105	< *0.001*^a^
Weight, kg^†^	50 (45–55) *n* = 109	60 (53–66) *n* = 106	< *0.001*^a^
BMI, mean (sd), kg/m^2^	19.3 (2.4) *n* = 109	19.8 (2.6) *n* = 105	0.136^a^
BMI < 18.5, number (%)	42 (38.5) (*n* = 109)	33 (31.4) (*n* = 105)	0.276^c^
Time elapsed between onset of symptoms and admittance to SPH, months, number (%)^d^			< *0.001*^b^
No symptoms^e^	6 (5.3)	14 (12.3)	–
< 1	17 (14.9)	37 (32.5)	–
1–6	68 (59.6)	49 (43.0)	–
6–12	12 (10.5)	8 (7.0)	–
> 12	11 (9.6)	6 (5.3)	–
Acid-fast bacilli, number (%)			< *0.001*^b^
1+	56 (49.1)	33 (28.9)	–
2+	29 (25.4)	28 (24.6)	–
3+	23 (20.3)	30 (26.3)	–
4+	6 (5.3)	23 (20.2)	–
Anti-TB antibody, number (%)	56 (49.1)	49 (43.0)	0.352^c^
T-SPOT.TB, number (%)	66 (84.62) *n* = 78	73 (90.12) *n* = 81	0.295^c^

*TB* tuberculosis. Italicized numbers indicated a *P* value of < 0.05

^†^Data are displayed as medians and interquartile ranges

^a^*t* tests

^b^Wilcoxon tests

^c^*χ*^2^ tests

^d^TB-correlated symptoms include cough, expectoration, fever, fatigue, hemoptysis, appetite loss, dyspnea, respiratory distress, insomnia, palpitation and weight loss

^e^Patients without typical TB symptoms; diagnosed by physical examination

Higher sputum bacterial counts (3+ and 4+) were observed in 46.5% of male patients compared to 25.6% in female patients. There were no statistical differences between male and female TB patients in the ratios of cases with positive responses in both the anti-TB antibody and the T-SPOT.TB tests (Table 1).

### Differential lung lesions and response to anti-TB treatment in male and female patients

Consistent with the results of the sputum bacterial counts, HRCT scans at T0 showed more severe lung damage in men with TB than in women with TB, represented by more cavitary lesions (70.5% versus 37.2%, *P* < 0.001), as well as healing-associated cicatricial emphysema, parenchymal bands, bronchovascular distortion and pleural thickening (Table 2).

**Table 2:** CT findings of study patients

CT findings	Prevalence of CT finding, no. (%)		*P* value^b^
	Female (*n* = 94)	Male (*n* = 95)	
Centrilobular nodules	93 (98.94)	94 (98.95)	1.000
Micronodules	91 (96.81)	93 (97.89)	0.682
Bronched nodule	88 (93.62)	89 (93.68)	0.985
Miliary nodule	1 (1.06)	0 (0.00)	0.497
Tree in bud	86 (91.49)	84 (88.42)	0.483
Cavity, number (%)	35 (37.2)	67 (70.5)	< *0.001*^a^
Thin-walled cavity	1 (1.10)	1 (1.10)	–
Thick-walled cavity	32 (34.00)	58 (61.10)	–
Both thin- and thick-walled cavity	2 (2.10)	8 (8.40)	–
Aspergillosis	0 (0.00)	3 (3.16)	0.246
Bronchial wall thickening	33 (35.11)	43 (45.26)	0.154
Bronchiectasis	27 (28.72)	38 (40.00)	0.103
Bronchial impaction	45 (47.87)	49 (51.58)	0.610
Emphysema (lobular)	1 (1.06)	9 (9.47)	*0.018*
Cicatricial emphysema	1 (1.06)	15 (15.79)	*0.001*
Bullae	0 (0.00)	5 (5.26)	0.059
Ground glass opacity	40 (42.55)	29 (30.53)	0.086
Consolidation	59 (62.77)	60 (63.16)	0.956
Atelectasis	6 (6.40)	1 (1.10)	0.065
Calcification	33 (35.10)	36 (7.90)	0.691
Mediastinal lymphadenopathy	13 (13.80)	20 (21.10)	0.191
Hilar lymphadenopathy	9 (9.60)	18 (18.90)	0.066
Bands (parenchymal)	20 (21.30)	37 (38.90)	*0.008*
Bronchiovascular distortion	7 (7.40)	20 (21.10)	*0.008*
Pleural thickening	60 (63.80)	74 (77.90)	*0.033*
Pleural effusion	3 (3.20)	4 (4.20)	1.000

*CT* computed tomography

^a^Wilcoxon tests

^b^*χ*^2^ tests

Italicized numbers indicate a *P* value of < 0.05

In the follow-up analysis of the overall response to the therapy, the grades of the bacterial loads in sputa showed a faster negative-conversion tendency (*P* = 0.069) in women (60.9%, 53/87) than in men (46.8%, 37/79) after 1 month of treatment (Additional file 1: Table S1).

Changes in the HRCT indices reflected detailed responses to standard anti-TB treatment. However, as fewer CT records (34 females and 24 males at T1 and 36 females and 28 males at T3) from the enrolled patients could be tracked back to T1 and T3 (Additional file 1: Table S2), the changes in CT images from patients with complete T0, T1 and T3 records were better to reflect the response to the treatment.

In 15 female and 7 male TB patients with complete HRCT records at T0, T1 and T3, the HRCT scores, which are results of simple addition of each index of the lung pathology in details, showed significant decrease in both male and female patients after 3 months of treatment, with a significant difference between the groups (*P* = 0.044), while only women patients showed a significant decrease in HRCT scores weighted by the presence of cavities (Table 3). Cavity results from accumulating immune response of the host after TB infection; therefore, the changes of weighted HRCT scores reflected the hosts’ responses to the treatment on the whole.

**Table 3:** HRCT scores in men and women with TB at differential time point of anti-TB treatment

		Female^†^ (*n* = 15)	Male^†^ (*n* = 7)
HRCT scores	T0	16.8 (11.23, 22.37)	25.86 (15.19, 36.53)
	T1	13.53 (8.45, 18.62)	23.86 (14.8, 32.91)
	T3	10.00 (4.89, 15.11)	18.71 (10.24, 27.19)
	*P* value^a^	< *0.001*	*0.001*
	*P* value^b^	*0.044*	
Weighted HRCT scores	T0	38.13 (23.51, 52.75)	48.71 (23.48, 73.95)
	T1	32.20 (17.48, 46.92)	52.43 (30.16, 74.69)
	T3	23.33 (9.54, 37.13)	41.57 (17.28, 65.86)
	*P* value^a^	*0.003*	0.295
	*P* value^b^	0.158	

*HRCT* high-resolution computed tomography, *T0* time of registration in SPH and before anti-TB treatment, *T1* about 1 month after T0, *T3* about 3 months after T0

^†^Data are displayed as means and lower and upper bounds of 95% confidence interval

^a^ANOVA tests with single-factor repeated measures compare the difference of scores at T0, T1 and T3 in female or male TB patients

^b^ANOVA tests with multiple-factor repeated measures compare the changes of scores at T0, T1 and T3 between female and male TB patients

Italicized numbers indicate a *P* value of < 0.05

### Critical indices associated with differential lung lesions between men and women with TB

To attempt to find the factors associated with more severe lung lesions which were influenced by sex, we firstly compared a total of 48 of the physiological, inflammatory, immunological (from CBC) and coagulation-associated clinical indices between the two groups, taking the CBC data from healthy men and women as baselines.

In healthy controls, men and women were mostly different in RBC-associated indices, with different ranges of normal values in RBC counts and HCT, and HGB levels; similarly, the values of these indices from men and women with TB were significantly different (Additional file 2: Figure S1).

**Fig. 1:**
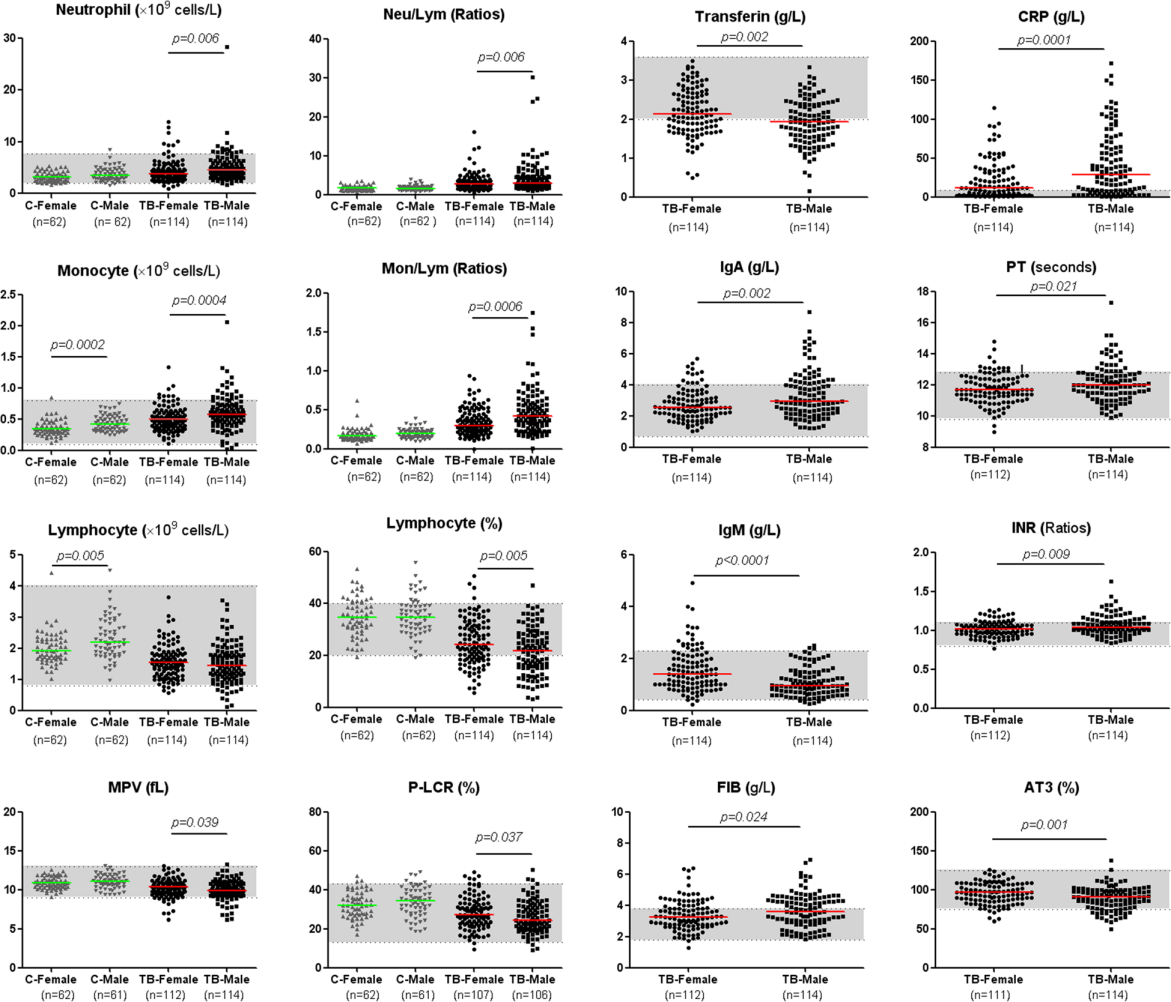
The indices with remarkable deviation from normal ranges and with significant differences between male and female TB patients. Horizontal lines represent median values. Grey areas represent the normal ranges of the indices. The differences between groups were analysed by Mann-Whitney *U* tests. MPV mean platelet volume, P-LCR platelet-large cell ratio, IgA immunoglobulin A, IgM immunoglobulin M, FIB fibrinogen, CRP C-reactive protein, PT prothrombin time, INR international normalized ratio, AT3 antithrombin III, Mon/Lym monocyte to lymphocyte ratio

There were significant differences between men and women in the counts of lymphocytes, monocytes and white blood cells in the healthy group. The differences of indices between men and women with TB were shown in Fig. 1 and Additional file 3: Figure S2.

Multivariate binary logistic regression (forward stepwise) was carried out to find out which differences in indices between men and women with TB were associated with severity of lung lesions. Although the indices which were selected in the models were different in separate male or female groups, and in combination, we found that similar sets of coagulation and platelet indices were involved in association with severity of male (MPV, AT3, FIB with sputum bacterial grade, PCT with cavity) and female (PT with cavity) patients with TB, and in combination of the groups (APTT with sputum bacterial grade, PCT with cavity) (Tables 4 and 5).

**Table 4:** Summary of the variables associated with sputum bacterial counts in the models derived from multivariate regression analysis with 48 indices and cases with only men (*n* = 114) or women (*n* = 114) or in combination (*n* = 228)

Variables in the equation	Sig.			OR			95% CI for OR					
							Lower			Upper		
	Combined	Male	Female	Combined	Male	Female	Combined	Male	Female	Combined	Male	Female
Sex (male)^a^	*0.006*	/	/	3.717	/	/	1.445	/	/	9.524	/	/
BMI	/	*0.012*	/	/	2.168	/	/	1.186	/	/	3.965	/
GLU (mmol/L)	0.052	/	/	2.447	/	/	0.992	/	/	6.031	/	/
HistoryofTB	*0.042*	0.086	/	0.000	0.000	/	0.000	0.000	/	0.000	0.000	/
HistoryofTB(1)	0.186	0.997	/	0.308	0.986	/	0.054	0.001	/	1.767	1653.893	/
HistoryofTB(2)	0.622	0.518	/	1.498	12.574	/	0.301	0.006	/	7.454	26,894.141	/
HistoryofTB(3)	0.142	0.912	/	0.191	0.645	/	0.021	0.000	/	1.739	1524.939	/
HistoryofTB(4)	0.401	0.139	/	0.345	0.001	/	0.029	0.000	/	4.140	8.486	/
Lym (× 10^9^/L)	*0.000*	*0.003*	/	0.083	0.002	/	0.024	0.000	/	0.290	0.120	/
MCHC (g/L)^c^	*0.019*	/	/	1.062	/	/	1.010	/	/	1.117	/	/
ESR (mm/h)^c^	*0.000*	/	*0.023*	1.036	/	1.023	1.017	/	1.003	1.055	/	1.043
APTT (s)	*0.009*	/	/	1.141	/	/	1.033	/	/	1.260	/	/
AT3 (%)	/	*0.020*	/	/	0.915	/	/	0.849	/	/	0.986	/
FIB (g/L)	/	*0.002*	/	/	15.968	/	/	2.879	/	/	88.572	/
MPV (fL)	/	*0.014*	/	/	0.181	/	/	0.046	/	/	0.709	/
TBAb(+)^b^	/	*0.006*	/	/	0.013	/	/	0.001	/	/	0.283	/

*BMI* body mass index, *GLU* fasting glucose, *Lym* counts of lymphocytes, *MCHC* mean corpuscular hemoglobin concentration, *ESR* erythrocyte sedimentation rate, *APTT* activated partial thromboplastin time, *AT3* antithrombin III, *FIB* fibrinogen, *MPV* mean platelet volume, *TBAb* anti-TB antibody response

ORs are calculated from binary logistic analysis (forward stepwise, conditional); italicized numbers indicate a *P* value of < 0.05

^a^Female as reference

^b^Cases with negative response as reference

^c^Indices with *P* < 0.05, but the value of OR ratio (95% CI) includes/ is close to 1.000

**Table 5:** Summary of the variables associated with cavity in the models of multivariate regression analysis with 48 indices and cases with only men (*n* = 114) or women (*n* = 114) or in combination (*n* = 228)

Variables in the equation	Sig.			OR			95% CI for OR					
							Lower			Upper		
	Combined	Male	Female	Combined	Male	Female	Combined	Male	Female	Combined	Male	Female
BMI	/	*0.004*	/	/	0.516	/	/	0.330	/	/	0.807	/
SputumSmear (1+)	*0.012*	/	/		/	/		/	/		/	/
SputumSmear(2+)	0.637	/	/	1.308	/	/	0.430	/	/	3.981	/	/
SputumSmear(3+)	*0.012*	/	/	5.913	/	/	1.480	/	/	23.621	/	/
SputumSmear(4+)	*0.007*	/	/	26.806	/	/	2.468	/	/	291.155	/	/
RDWCV	*0.001*	/	/	2.509	/	/	1.464	/	/	4.302	/	/
HGB (g/L)	/	/	*0.009*	/	/	0.928	/	/	0.877	/	/	0.981
CRP (mg/L)^a^	*0.000*	/	/	1.043	/	/	1.020	/	/	1.068	/	/
ESR (mm/h)^a^	/	*0.010*	/	/	1.076	/	/	1.018	/	/	1.138	/
PCT (%)	*0.000*	*0.004*	/	0.000	0.000	/	0.000	0.000	/	0.000	0.000	/
PT (s)	/	/	*0.046*	/	/	2.242	/	/	1.016	/	/	4.951
C4 (g/L)	/	*0.016*	/	/	1.34E+10	/	/	75.786	/	/	2.38E+18	/
TBAb(+)^b^	*0.008*	/	/	4.372	/	/	1.469	/	/	13.010	/	/

*BMI* body mass index, *RDW-CV* red blood cell distribution width-coefficient of variation, *HGB* haemoglobin, *CRP* C-reactive protein, *ESR* erythrocyte sedimentation rate, *PCT* plateletcrit, *PT* prothrombin time, *C4* complement component 4, *TBAb* anti-TB antibody response

ORs are calculated from binary logistic analysis (forward stepwise, conditional); italicized numbers indicated a* P* value of <0.05

^a^Indices with *P* value < 0.05 but OR ratio close to 1

^b^Indices with *P* < 0.05, but the value of OR ratio (95% CI) includes/is close to 1.000

**Fig. 2:**
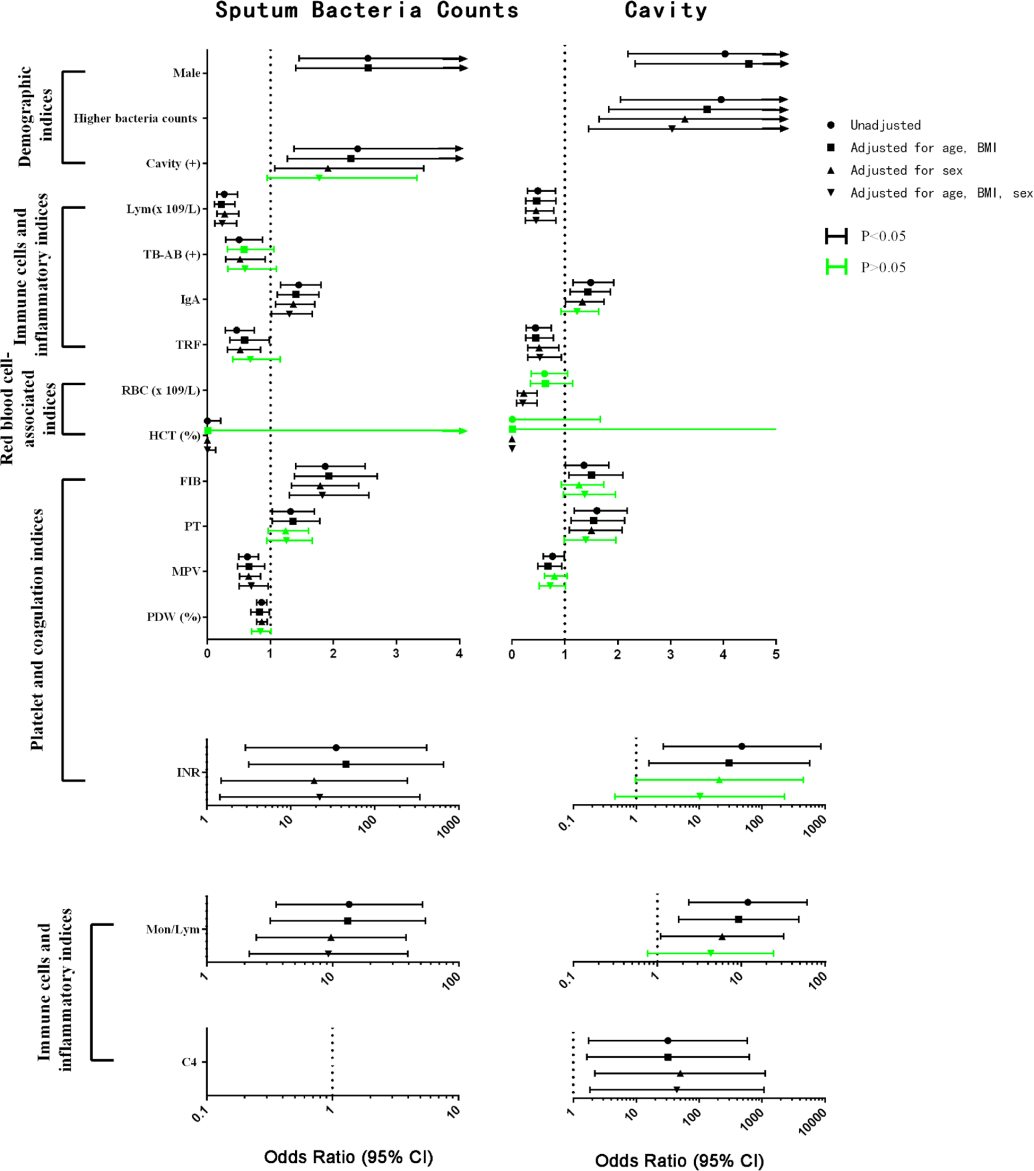
Forest plot of the indices associated with sputum bacteria counts (left) and cavity (right) among 48 indices in 228 TB patients. The dots show the unadjusted odds ratios of the association of the indices with sputum bacteria counts or cavity. Small squares indicate ORs after being adjusted with age and BMI; small triangles indicate ORs after being adjusted with sex; inverted small triangles indicate ORs after being adjusted with age, BMI and sex. Lines represent the values of the 95% CI. Black lines depict *P* < 0.05. Green lines depict *P* > 0.05 after adjustment

In univariate regression analysis, higher grades of sputum bacterial counts were associated with increased incidence of cavities. As in a previous report [17], male sex is a predisposing factor for higher grades of sputum bacterial counts and presence of cavities, independent of age and BMI (Fig. 2 and Additional file 1: Table S3-S6). Besides, lower lymphocyte counts were associated with increased sputum bacterial counts and presence of cavities, independent of sex, age and BMI.

Other indices associated with both sputum bacterial counts and cavities were monocyte to lymphocyte ratio, IgA and TRF levels, and platelet- and coagulation-associated indices: MPV, PT, INR and FIB. These associations were influenced by adjustment with sex or age and BMI, separately or in combination. The association between platelet and coagulation indices with either cavity (MPV, FIB and INR) or cavity-associated sputum bacterial grade (PT) was mostly influenced by sex adjustment (Fig. 2 and Additional file 1: Table S3-S6).

## Discussion

Based on comparison of the severity of lung lesions between TB with diabetes and TB without diabetes, our previous study demonstrated that men with TB are associated with larger areas of necrosis in granuloma, and haemostasis indices signified exacerbated lung lesions, even at the early stage of TB [17]. To exclude the potential influence of the indices with men’s tendency, e.g. smoking or other confounding factors in the previous research, this study was carried out with well-matched non-smoking men and women with primary TB without comorbidities. Our study provides clinical evidence that men are associated with more cavities and higher sputum bacterial counts than women at the time of first TB registration [18].

Previous reports indicated that severe TB correlates with neutrophil abundance and lymphocyte deficiency [19, 20]. In our study, lower counts of peripheral lymphocytes were associated with both higher sputum bacterial loads and cavity in primary TB. Peripheral neutrophils, which were associated with sputum bacteria counts in the study involving more severe TB cases (e.g. TB with diabetes comorbidity) [17], were not associated with either cavity or sputum bacterial counts in this study.

As we have simplified our study by excluding patients with confounding factors, like smoking [21], that may influence the pathogenesis of TB, the significant difference between men and women in the severity of TB-induced lung lesions may reflect the overall influences of differential sexual hormones, sex-related genetic backgrounds, genetic regulation and metabolism on the basic immune response and susceptibility to infection of *Mtb*.

Women have a better ability to clear pathogens [22], and sex steroid immunomodulation has been linked to higher infection rates in men. Androgens influence gut microbiota [23], which may provoke host susceptibility to TB [24]. Estrogen promotes and testosterone downregulates T-helper (Th)1 cells, macrophage activation and antibody-dominated responses [8, 25], which are considered to play important roles in tuberculosis immune responses [26].

However, in the T-SPOT.TB and serum anti-TB IgG tests, similar ratios of cases with positive response were found in male and female patients. These results indicated that there were no significant differences in the frequency of TB antigen (ESAT-6, CFP-10)-specific memory T cells and TB antigen (lipoarabinomannan and 38KD antigen)-specific antibody responses between the groups; the latter two might not be the main contributors for male bias in TB pathogenesis either.

Although we did not have all information about the baseline differences in all the indices between men and women, regression analysis may give indications for the associated factors with extensive lung injury in man with TB on the whole, which include the associated factors with sex bias derived from either differential physical background or differential response to TB infection.

Among all the 48 physiological, immunological and inflammatory indices, platelet and coagulation indices are identified as the associated cluster of indices with the lung lesions from both the univariate and multivariate logistic regressions, with either separate or combination cases of men and women. Consistently, the clinical manifestations of TB included systemic hypercoagulable states [27], and severe pulmonary TB was complicated by deep vein thrombosis in some cases [28]. Our previous study revealed that activation of coagulation and platelets (shortened PT and increased PDW) is associated with aggravated caseous necrosis and concomitant severe fibroplasia in granulomas, even at the early stage of TB pathogenesis (TB patients with negative sputum smear results and restricted lung lesion area) [17]. In this study, however, in TB cases with positive sputum smear results, increased PT and PT-associated INR are associated with more severe lung lesions (higher grades of sputum bacterial counts and presence of cavities). Similar reversion of the association in patients at different stages of TB disease also occurs between platelet indices (MPV and PDW) and the lung lesions [17]. Dynamic activation of the platelets and coagulation pathway occurs with TB infection. Increased INR and PT and reduced MPV and PDW values may reflect the exhaustion of the peripheral coagulation and platelets in patients with open lesions during the host combating with *Mtb*. infection. Consistently, recent research indicated that platelets drive a proinflammatory, tissue-degrading phenotype in TB [29]. The results not only substantiate the hypothesis that platelets and coagulation pathway play important roles in TB infection, but also indicate that the association between these haemostasis indices and lung lesions is influenced mostly by sex.

Although there is a dearth of information regarding the mechanism of the sexual dimorphism of the incidence and outcome of the coagulation-associated disease (e.g. stroke) [30], it is known that genes coding for coagulation factors VIII and IX are located on chromosome X; moreover, gonadal hormone exposure can impact coagulation and fibrinolysis [30]. Reactivity of human platelets may be influenced by sex and sex hormones, as both men and women express estrogen receptors on their platelets [31]. Some but not all reports [32] show that testosterone may activate [33] while progesterone and 17-beta estradiol may inhibit [34] aggregation of human platelets. However, differential basal expression of these coagulation factors between men and women and differential active status of the coagulation regulated by sex hormones during infection may result in different immune response and susceptibility and therefore sex bias in TB pathogenesis.

The lesser severity of TB lesions in women than in men may not only explain the male bias in the incidence of TB, but also correlate with a longer delay in seeking hospital treatment in female TB patients as shown in our investigation and in a previous report [35]. The sex bias in TB pathogenesis may also correlate with delayed recovery responses to anti-TB treatment in male patients. Therefore, studies on sex-associated disparities, especially on infection-correlated activation of coagulation and other critical modulating mechanisms involved in TB pathogenesis, may help to better understand the heterogeneity that is intrinsic to TB at the population level [36]; these studies will be crucial for better adaptation of future intervention strategies at the community level [18] and to inform the development of intervention strategies for severe cases of TB in male patients.

We should point out some of the shortcomings in this study. We have no precise information of the socioeconomic background and therefore nutritional status of patients before their registration; the latter is a key factor which influences the infected person progressing from latent TB to active TB [37]. However, as there were no statistical differences between men and women both in their BMI indices and in their ratio of cases with malnutrition (cases with BMI < 18.5), we therefore regard that the nutritional status between men and women with TB are similar and therefore have no critical impact on the sex bias in the pathogenesis of TB. Another shortcoming is that, although we have found that the association of the haemostasis with the lung lesions in TB is mostly influenced by sex, we still do not know the precise underlying molecular mechanism. Moreover, although there is no association of counts of peripheral neutrophils with lung lesions, difference in counts of neutrophil was found between male and female TB patients. Therefore, we cannot exclude the possibility of differential levels of neutrophil-derived soluble factors, such as matrix metalloproteinases [38, 39], driving differential cavity formation in male and female TB patients. Works based on mouse models may help to clarify the critical factor(s) involved in the sex bias in TB pathogenesis.

Furthermore, in multivariate analysis of independent associated indices for lung lesions, higher sputum bacterial counts are associated with cavitation, but not vice versa. Our result is consistent with a recent report that in those cases without cavitation, the radiological severity of disease on chest X-ray prior to treatment in smear-positive pulmonary TB patients has no association with the bacterial burden [40]. It may therefore explain the seemingly paradox association of BMI [41–43] and anti-TB antibody [26, 44, 45] response with the bacterial counts and cavity in TB patients, in our data and previous reports.

## Conclusions

Our study provides strong evidence that men with primary TB have significantly more severe lung lesions than women and that differential basal or infection-induced coagulation dysfunction may be involved in TB pathogenesis.

### Perspectives and significance

Our findings that the association between coagulation and lung injury is influenced by gender not only suggest new mechanisms for the pathogenesis of tuberculosis, but also provide new insights into the biological basis of male-biased tuberculosis and other infectious diseases. An in-depth understanding of the role of the coagulation pathway in the development of these gender-biased diseases will facilitate our precise treatment and preventive measures for these diseases.

## Additional files

Additional file 1: **Table S1.** Time to negative conversion of sputum bacteria in female and male patients with TB. **Table S2.** Changes of CT findings in men and women with TB after differential time of anti-TB treatment. **Table S3.** Association of immune, biochemical indices and CT findings with sputum bacteria counts in 228 TB patients. **Table S4.** Association of immune, biochemical indices with cavity in 228 TB patients. **Table S5.** Association of immune, biochemical indices and CT findings with high sputum smear (3+ and 4+) in 228 TB patients. **Table S6.** Association of immune, biochemical indices with cavity in 228 TB patients. (DOCX 70 kb)

Additional file 2: **Figure S1.** Levels of red blood cell-associated indices in male and female healthy control and TB patients. Horizontal lines represent median values. Grey areas represent the normal ranges of the index in women; the area between dashed lines represent the normal ranges of the index in men. The differences between groups were analysed by Mann-Whitney *U* tests. RBC, red blood cell; HGB, haemoglobin; HCT, haematocrit; MCHC, mean corpuscular hemoglobin concentration. (TIF 2118 kb)

Additional file 3: **Figure S2.** The indices with no or negligible differences between male and female TB patients. Horizontal lines represent median values. Grey areas represent the normal ranges of the indices. The differences between groups were analysed by Mann-Whitney *U* tests. WBC, white blood cell; PCT, plateletcrit; APTT, activated partial thromboplastin time; C3, complement 3; C4, complement 4; FDP, fibrinogen degradation product; IgG, immunoglobulin G. (TIF 7818 kb)

## Abbreviations

AFB: Acid-fast bacilli
BMI: Body mass index
CBC: Complete blood count
FIB: Fibrinogen
HCT: Haematocrit
HRCT: High-resolution computed tomography
INR: International normalized ratios
MPV: Mean platelet volume
PCT: Plateletcrit
PDW: Platelet distribution width
PT: Prothrombin time
TB: Tuberculosis
TRF: Transferrin
