# Supplementary material for: Sex influences the association between haemostasis and the extent of lung lesions in tuberculosis
Source: Biol Sex Differ. 2018 Oct 10;9:44. doi: 10.1186/s13293-018-0203-9 (PMC6180492; doi:10.1186/s13293-018-0203-9)
Supplement: Supplementary file 1 — Table S1. Time to negative conversion of sputum bacteria in female and male patients with TB. Table S2. Changes of CT findings in men and women with TB after differential time of anti-TB treatment. Table S3. Association of immune, biochemical indices and CT findings with sputum bacteria counts in 228 TB patients. Table S4. Association of immune, biochemical indices with cavity in 228 TB patients. Table S5. Association of immune, biochemical indices and CT findings with high sputum smear (3+ and 4+) in 228 TB patients. Table S6. Association of immune, biochemical indices with cavity in 228 TB patients. (DOCX 70 kb) [file 13293_2018_203_MOESM1_ESM.docx]

**Table S1 Time to negative conversion of sputum bacteria in female and male patients with TB.**

| Time to negative conversion | Female (n=87) | Male (n=79) |
| --- | --- | --- |
| ≤1 Mon | 53 (60.9%) | 37 (46.8%) |
| >1 Mon | 34 (39.1%) | 42 (53.2%) |
| *P* | 0.069 ^a^ | |

**Table notes**: Data shown were presented as No. (%)；^a^ χ2 tests.

**Table S2 Changes of CT findings in men and women with TB after differential time of anti-TB treatment**

|  | **Female** | | |  | **Male** | | |
| --- | --- | --- | --- | --- | --- | --- | --- |
| **CT findings** | **T0**  **(n=94)** | **T1**  **(n=34)** | **T3**  **(n=36)** |  | **T0**  **(n=95)** | **T1**  **(n=24)** | **T3**  **(n=28)** |
| Centrilobular nodules | 93 (98.94) | 33 (97.06) | 34 (94.44) |  | 94 (98.95) | 24 (100.00) | 26 (92.86) |
| Micronodules | 91 (96.81) | 33 (97.06) | 30(83.33) |  | 93 (97.89) | 24 (100.00) | 25 (89.29) |
| Bronched nodule | 88 (93.62) | 33 (97.06) | 27 (75.00) |  | 89 (93.68) | 24 (100.00) | 25 (89.29) |
| Miliary nodule | 1 (1.06) | 1 (2.94) | 1 (2.78) |  | 0 (0.00) | 0 (0.00) | 0 (0.00) |
| Tree in bud | 86 (91.49) | 31 (91.18) | 23 (63.89) |  | 84 (88.42) | 24 (100.00) | 20 (71.43) |
| Cavity, number (%) | 35 (37.2) | 15 (44.12) | 8 (22.22) |  | 67 (70.53) | 18 (75.00) | 11 (39.29) |
| Thin walled cavity | 1 (1.10) | 1 (2.94) | 1 (2.78) |  | 1 (1.10) | 1 (4.17) | 0 (0.00) |
| Thick walled cavity | 32 (34.00) | 13 (38.24) | 7 (19.44) |  | 58 (61.10) | 12 (50.00) | 11 (39.29) |
| Both thin and thick-walled cavity | 2 (2.10) | 1 (2.94) | 0 (0.00) |  | 8 (8.40) | 5 (20.83) | 0 (0.00) |
| Aspergillosis | 0 (0.00) | 0 (0.00) | 0 (0.00) |  | 3 (3.16) | 0 (0.00) | 1 (3.57) |
| Bronchial wall thickening | 33 (35.11) | 13 (38.24) | 12 (33.33) |  | 43 (45.26) | 13 (54.17) | 10 (35.71) |
| Bronchiectasis | 27 (28.72) | 11 (32.35) | 11 (30.56) |  | 38 (40.00) | 12 (50.00) | 10 (35.71) |
| Bronchial impaction | 45 (47.87) | 18 (52.94) | 17 (47.22) |  | 49 (51.58) | 15 (62.50) | 11 (39.29) |
| Emphysema (lobular) | 1 (1.06) | 0 (0.00) | 1 (2.78) |  | 9 (9.47) | 3 (12.50) | 3 (10.71) |
| Cicatricial emphysema | 1 (1.06) | 0 (0.00) | 0 (0.00) |  | 15 (15.79) | 3 (12.50) | 3 (10.71) |
| Bullae | 0 (0.00) | 0 (0.00) | 0 (0.00) |  | 5 (5.26) | 2 (8.33) | 1 (3.57) |
| Ground glass opacity | 40 (42.55) | 18 (52.94) | 6 (16.67) |  | 29 (30.53) | 10 (41.67) | 10 (35.71) |
| Consolidation | 59 (62.77) | 24 (70.59) | 14 (38.89) |  | 60 (63.16) | 17 (70.83) | 15 (53.57) |
| Atelectasis | 6 (6.40) | 6 (17.65) | 2 (2.56) |  | 1 (1.10) | 0 (0.00) | 0 (0.00) |
| Calcification | 33 (35.10) | 16 (47.06) | 11 (30.56) |  | 36 (37.90) | 6 (25.00) | 7 (25.00) |
| Mediastinal lymphadenopathy | 13 (13.80) | 7 (20.59) | 4 (11.11) |  | 20 (21.10) | 8 (33.33) | 3 (10.71) |
| Hilar lymphadenopathy | 9 (9.60) | 7 (20.59) | 2 (2.56) |  | 18 (18.90) | 7 (29.17) | 3 (10.71) |
| Bands (parenchymal) | 20 (21.30) | 9 (26.47) | 6 (16.67) |  | 37 (38.90) | 10 (41.67) | 11 (39.29) |
| Bronchiovascular distortion | 7 (7.40) | 3 (8.82) | 1 (2.78) |  | 20 (21.10) | 6 (25.00) | 3 (10.71) |
| Pleural thickening | 60 (63.80) | 26 (76.47) | 21 (58.33) |  | 74 (77.90) | 20 (83.33) | 22 (78.57) |
| Pleural effusion | 3 (3.20) | 3 (8.82) | 1 (2.78) |  | 4 (4.20) | 2 (8.62) | 0 (0.00) |

**Table notes**: Data shown were presented as No. (%). T0, time of registration in SPH and before anti-TB treatment; T1, about 1 month after T0; T3, about 3 months after T0.

Table S3 Association of immune, biochemical indices and CT findings with sputum bacteria counts in 228 TB patients.

| **Parameters** | **Unadjusted** | | | **Adjusted for age, BMI** | | | | **Adjusted for sex** | | | | | **Adjusted for sex, age, BMI** | | | | |  |
| --- | --- | --- | --- | --- | --- | --- | --- | --- | --- | --- | --- | --- | --- | --- | --- | --- | --- | --- |
|  | **OR** | **( 95% CI )** | ***P*** | | **OR** | **( 95% CI )** | ***P*** | | **OR** | **( 95% CI )** | | ***P*** | | **OR** | **( 95% CI )** | | ***P*** | |
| Male^a^ | 2.55 | (1.46, 4.46) | **0.001** | | 2.55 | (1.40, 4.64) | **0.002** | | - | - |  | - | | - | - |  | - | |
| Cavity | 2.39 | (1.37, 4.15) | **0.002** | | 2.28 | (1.27, 4.10) | **0.006** | | 1.92 | (1.07, 3.43) | | **0.029** | | 1.78 | (0.95, 3.33) | | 0.073 | |
| INR | 34.81 | (2.90, 417.22) | **0.005** | | 45.80 | (3.18, 660.54) | **0.005** | | 19.11 | (1.48, 246.31) | | **0.024** | | 22.23 | (1.43, 346.01) | | **0.027** | |
| Mon/Lym | 13.53 | (3.55, 51.62) | **0.000** | | 13.20 | (3.19, 54.63) | **0.000** | | 9.74 | (2.48, 38.26) | | **0.001** | | 9.28 | (2.17, 39.59) | | **0.003** | |
| FIB | 1.87 | (1.40, 2.51) | **0.000** | | 1.93 | (1.38, 2.69) | **0.000** | | 1.79 | (1.34, 2.40) | | **0.000** | | 1.83 | (1.30, 2.57) | | **0.000** | |
| IgA (g/L) | 1.45 | (1.16, 1.80) | **0.001** | | 1.40 | (1.11, 1.78) | **0.005** | | 1.36 | (1.08, 1.71) | | **0.008** | | 1.30 | (1.01, 1.66) | | **0.039** | |
| PT (seconds) | 1.32 | (1.03, 1.70) | **0.030** | | 1.36 | (1.03, 1.79) | **0.030** | | 1.24 | (0.96, 1.61) | | 0.101 | | 1.25 | (0.94, 1.66) | | 0.120 | |
| Lym(×10^9^/L) | 0.27 | (0.15, 0.48) | **0.000** | | 0.22 | (0.11, 0.44) | **0.000** | | 0.28 | (0.15, 0.49) | | **0.000** | | 0.23 | (0.12, 0.46) | | **0.000** | |
| MPV (fL) | 0.64 | (0.50, 0.81) | **0.000** | | 0.66 | (0.48, 0.91) | **0.011** | | 0.66 | (0.51, 0.84) | | **0.001** | | 0.70 | (0.50, 0.96) | | **0.029** | |
| PDW (%) | 0.86 | (0.78, 0.94) | **0.001** | | 0.83 | (0.69, 0.98) | **0.031** | | 0.86 | (0.79, 0.95) | | **0.002** | | 0.84 | (0.70, 1.01) | | 0.057 | |
| TRF (g/L) | 0.46 | (0.29, 0.75) | **0.001** | | 0.59 | (0.36, 0.98) | **0.042** | | 0.52 | (0.32, 0.85) | | **0.008** | | 0.68 | (0.40, 1.15) | | 0.153 | |
| HCT (%) | 0.00 | (0.00, 0.21) | **0.013** | | 0.01 | (0.00, 7.96) | 0.174 | | 0.00 | (0.00, 0.00) | | **0.000** | | 0.00 | (0.00, 0.13) | | **0.013** | |
| TB-Ab^b^ | 0.50 | (0.29, 0.88) | **0.016** | | 0.58 | (0.32, 1.05) | 0.074 | | 0.52 | (0.29, 0.91) | | **0.023** | | 0.60 | (0.33, 1.10) | | 0.095 | |

**Table notes:** ORs are calculated from binary logistic analysis. Patients were divided into two groups according to low (1+ and 2+) and high (3+ and 4+) sputum bacteria counts. Bold numbers indicate a *P* value of < 0.05. The backgrounds of data (Odd ratios, 95% CI and the *P* values) were highlighted with dark grey (*P* >0.05).

INR=international normalized ratios; Mon/ Lym=monocyte to lymphocyte ratio; FIB=fibrinogen; IgA=immunoglobulin A; PT= prothrombin time; Lym (×10^9^/L)=counts of lymphocyte; MPV= Mean platelet volume; PDW=Platelet distribution width; TRF=transferrin; HCT=hematocrit; TB-Ab=anti-TB IgG antibody.

^a^ female as the reference. ^b^ patients with negative results of anti-TB antibody as the reference.

Table S4 Association of immune, biochemical indices with cavity in 228 TB patients.

| **Parameters** | **Unadjusted** | | | | **Adjusted for age, BMI** | | | | **Adjusted for sex** | | | **Adjusted for sex, age, BMI** | | | |
| --- | --- | --- | --- | --- | --- | --- | --- | --- | --- | --- | --- | --- | --- | --- | --- |
|  | **OR** | **( 95% CI )** | ***P*** | **OR** | | **( 95% CI )** | ***P*** | **OR** | | **( 95% CI )** | ***P*** | | **OR** | **( 95% CI )** | ***P*** |
| Male^a^ | 4.03 | (2.20, 7.41) | **0.000** | 4.48 | | (2.33, 8.63) | **0.000** | - | | - | - | | - | - | - |
| Higher bacteria counts | 3.96 | (2.05, 7.64) | **0.000** | 3.69 | | (1.83, 7.42) | **0.000** | 3.27 | | (1.65, 6.49) | **0.001** | | 3.03 | (1.45, 6.31) | **0.003** |
| INR | 48.07 | (2.68, 863.21 | **0.009** | 30.15 | | (1.60, 568.51) | **0.023** | 20.99 | | (0.98, 450.45) | 0.052 | | 10.28 | (0.46, 229.61) | 0.141 |
| C4 (g/L) | 31.94 | (1.74, 586.58) | **0.020** | 32.15 | | (1.65, 626.89) | **0.022** | 50.09 | | (2.22, 1132.41) | **0.014** | | 44.25 | (1.83, 1069.16) | **0.020** |
| Mono/Lym | 12.02 | (2.37, 61.02) | **0.003** | 9.34 | | (1.80, 48.35) | **0.008** | 5.92 | | (1.10, 31.81) | **0.038** | | 4.32 | (0.77, 24.21) | 0.096 |
| IgA (g/L) | 1.49 | (1.15, 1.92) | **0.002** | 1.43 | | (1.10, 1.87) | **0.008** | 1.33 | | (1.02, 1.75) | **0.038** | | 1.23 | (0.93, 1.64) | 0.152 |
| PT (seconds) | 1.61 | (1.18, 2.18) | **0.002** | 1.55 | | (1.12, 2.13) | **0.008** | 1.50 | | (1.09, 2.09) | **0.014** | | 1.40 | (0.99, 1.97) | 0.056 |
| FIB (g/L) | 1.36 | (1.02, 1.83) | **0.040** | 1.50 | | (1.08, 2.10) | **0.017** | 1.27 | | (0.93, 1.73) | 0.129 | | 1.38 | (0.97, 1.96) | 0.071 |
| MPV (fL) | 0.77 | (0.60, 0.99) | **0.039** | 0.68 | | (0.49, 0.95) | **0.021** | 0.80 | | (0.62, 1.05) | 0.105 | | 0.72 | (0.51, 1.02) | 0.062 |
| Lym (×10^9^/L) | 0.49 | (0.29, 0.82) | **0.007** | 0.47 | | (0.27, 0.83) | **0.009** | 0.46 | | (0.26, 0.79) | **0.005** | | 0.46 | (0.25, 0.84) | **0.011** |
| TRF (g/L) | 0.45 | (0.27, 0.75) | **0.002** | 0.45 | | (0.26, 0.78) | **0.005** | 0.52 | | (0.30, 0.88) | **0.016** | | 0.53 | (0.30, 0.94) | **0.029** |

**Table notes**: ORs are calculated from binary logistic analysis. Bold numbers indicate a P value of < 0.05. The backgrounds of data (Odd ratios, 95% CI and the *P* values) were highlighted with dark grey (*P* >0.05). C4= complement component 4; See Supplementary Table 3 legends for expansion of other abbreviation.

^a^ female as reference.

**Table S5 Association of immune, biochemical indices and CT findings with high sputum smear (3+ & 4+) in 228 TB patients.**

| **Parameters** | **Unadjusted** | | | **Adjusted for age, BMI** | | | **Adjusted for sex** | | | **Adjusted for sex, age, BMI** | |
| --- | --- | --- | --- | --- | --- | --- | --- | --- | --- | --- | --- |
|  | **OR (95% CI)** | ***P*** | **OR (95% CI)** | | ***P*** | **OR (95% CI)** | | ***P*** | **OR (95% CI)** | | ***P*** |
|  |  |  |  |  |  |  |  |  |  |  |  |
| **Demographic indices** | | |  | |  |  | |  |  | |  |
| Age (year) | 1.02 (1.00, 1.04) | 0.114 | - | | - | 1.02 (1.00, 1.04) | | 0.102 | - | | - |
| Weight (kg) ^b^ | 0.99 (0.96, 1.02) | 0.648 | 1.04 (0.99, 1.10) | | 0.144 | 0.95 (0.92, 0.99) | | **0.017** | 0.95 (0.87, 1.03) | | 0.174 |
| Height (cm) | 1.02 (0.99, 1.06) | 0.234 | 1.03 (0.99, 1.07) | | 0.111 | 0.96 (0.91, 1.01) | | 0.125 | 0.97 (0.92, 1.02) | | 0.233 |
| BMI | 0.92 (0.82, 1.04) | 0.180 | - | | - | 0.90 (0.80, 1.02) | | 0.095 | - | | - |
| T-spot ^a^ | 1.00 (0.99, 1.01) | 0.596 | 1.00 (0.99, 1.01) | | 0.937 | 1.00 (0.99, 1.01) | | 0.584 | 1.00 (0.99, 1.01) | | 0.971 |
| **Red blood cell-associated indices** | | | | |  |  | |  |  | |  |
| RBC(×10^12^/L) | 0.67 (0.40, 1.10) | 0.109 | 0.93 (0.53, 1.65) | | 0.807 | 0.45 (0.25, 0.78) | | 0.005 | 0.64 (0.34, 1.20) | | 0.164 |
| HGB (g/L) ^b^ | 0.98 (0.97, 1.00) | **0.022** | 0.99 (0.97, 1.01) | | 0.188 | 0.97 (0.95, 0.99) | | **0.000** | 0.98 (0.96, 1.00) | | **0.014** |
| MCH (pg) | 0.92 (0.82, 1.04) | 0.172 | 0.91 (0.80, 1.03) | | 0.121 | 0.91 (0.80, 1.02) | | 0.105 | 0.90 (0.79, 1.02) | | 0.099 |
| MCHC (g/L) | 1.00 (0.97, 1.02) | 0.807 | 1.00 (0.97, 1.02) | | 0.731 | 0.99 (0.96, 1.01) | | 0.345 | 0.99 (0.96, 1.02) | | 0.452 |
| MCV (fL) | 0.97 (0.92, 1.01) | 0.138 | 0.96 (0.91, 1.01) | | 0.095 | 0.97 (0.92, 1.01) | | 0.146 | 0.96 (0.91, 1.01) | | 0.107 |
| RDW-CV (%) ^b^ | 1.24 (1.03, 1.50) | **0.024** | 1.18 (0.97, 1.44) | | 0.102 | 1.24 (1.03, 1.51) | | **0.026** | 1.17 (0.95, 1.44) | | 0.139 |
| RDW-SD (fL) | 1.05 (0.98, 1.12) | 0.183 | 1.02 (0.95, 1.09) | | 0.684 | 1.04 (0.98, 1.12) | | 0.196 | 1.01 (0.94, 1.09) | | 0.800 |
| **Platelet and coagulation indices** | | | | |  |  | |  |  | |  |
| P-LCR (%) ^b^ | 0.94 (0.91, 0.98) | **0.003** | 0.95 (0.91, 0.98) | | **0.005** | 0.95 (0.91, 0.99) | | **0.007** | 0.95 (0.92, 0.99) | | **0.016** |
| PLT(×10^9^/L) ^b^ | 1.00 (1.00, 1.01) | **0.027** | 1.00 (1.00, 1.01) | | 0.067 | 1.00 (1.00, 1.01) | | **0.034** | 1.00 (1.00, 1.01) | | 0.090 |
| PCT (%) | 3.29 (0.13, 84.16) | 0.471 | 6.69 (0.22, 208.20) | | 0.279 | 4.32 (0.16, 116.40) | | 0.384 | 7.75 (0.23, 259.80) | | 0.253 |
| APTT (s) ^b^ | 1.09 (1.03, 1.15) | **0.004** | 1.09 (1.03, 1.16) | | **0.005** | 1.08 (1.02, 1.14) | | **0.012** | 1.08 (1.02, 1.15) | | **0.013** |
| AT3 (%) ^b^ | 0.96 (0.94, 0.98) | **0.000** | 0.97 (0.94, 0.99) | | **0.001** | 0.97 (0.95, 0.99) | | **0.001** | 0.97 (0.95, 0.99) | | **0.006** |
| TT (s) | 0.89 (0.79, 1.01) | 0.059 | 0.90 (0.79, 1.02) | | 0.099 | 0.91 (0.80, 1.03) | | 0.130 | 0.92 (0.80, 1.05) | | 0.191 |
| D-Dimer (ng/mL) ^b^ | 1.00 (1.00, 1.00) | **0.022** | 1.00 (1.00, 1.00) | | 0.055 | 1.00 (1.00, 1.00) | | 0.050 | 1.00 (1.00, 1.00) | | 0.125 |
| FDP (µg/mL) ^b^ | 1.06 (1.01, 1.12) | **0.031** | 1.05 (1.00, 1.11) | | 0.067 | 1.05 (1.00, 1.11) | | **0.045** | 1.04 (0.99, 1.10) | | 0.108 |
| C4 (g/L) | 8.78 (0.63, 123.20) | 0.107 | 18.08 (1.11, 294.40) | | **0.042** | 7.92 (0.52, 120.90) | | 0.137 | 15.46 (0.87, 274.90) | | 0.062 |
| C3 (g/L) | 2.01 (0.78, 5.19) | 0.151 | 3.53 (1.23, 10.14) | | **0.019** | 2.02 (0.77, 5.32) | | 0.156 | 3.49 (1.18, 10.28) | | **0.024** |
| **Immune cells and inflammatory indices** | | | | | |  | |  |  | |  |
| BASO (%) | 0.81 (0.32, 2.09) | 0.667 | 0.70 (0.24, 2.03) | | 0.516 | 0.87 (0.33, 2.27) | | 0.773 | 0.79 (0.27, 2.33) | | 0.669 |
| EO (%) | 0.93 (0.80, 1.08) | 0.318 | 0.92 (0.78, 1.09) | | 0.341 | 0.92 (0.79, 1.08) | | 0.315 | 0.93 (0.78, 1.10) | | 0.398 |
| Lym (%) ^b^ | 0.94 (0.91, 0.97) | **0.000** | 0.94 (0.90, 0.97) | | **0.001** | 0.94 (0.91, 0.98) | | **0.001** | 0.95 (0.91, 0.98) | | **0.003** |
| Mon (%) ^b^ | 1.11 (1.00, 1.23) | **0.047** | 1.13 (1.01, 1.26) | | **0.029** | 1.11 (1.00, 1.23) | | 0.062 | 1.13 (1.01, 1.26) | | **0.031** |
| Neu (%) ^b^ | 1.04 (1.02, 1.07) | **0.002** | 1.04 (1.01, 1.07) | | **0.008** | 1.04 (1.01, 1.07) | | **0.006** | 1.04 (1.00, 1.07) | | **0.025** |
| BASO(×10^9^/L) | 0.00 (0.00, 385.70) | 0.242 | 0.00 (0.00, 20.34) | | 0.106 | 0.00 (0.00, 545.40) | | 0.256 | 0.00 (0.00, 58.35) | | 0.132 |
| EO(×10^9^/L) | 0.44 (0.05, 3.72) | 0.447 | 0.22 (0.02, 2.88) | | 0.250 | 0.35 (0.04, 3.18) | | 0.350 | 0.19 (0.01, 2.71) | | 0.219 |
| Mon(×10^9^/L) | 1.89 (0.63, 5.61) | 0.254 | 1.91 (0.60, 6.11) | | 0.273 | 1.30 (0.42, 4.01) | | 0.643 | 1.37 (0.41, 4.55) | | 0.609 |
| Neu(×10^9^/L) | 1.02 (0.92, 1.13) | 0.763 | 1.00 (0.90, 1.12) | | 0.976 | 0.99 (0.89, 1.10) | | 0.879 | 0.98 (0.87, 1.10) | | 0.696 |
| WBC(×10^9^/L) | 0.97 (0.87, 1.07) | 0.519 | 0.95 (0.85, 1.07) | | 0.390 | 0.94 (0.84, 1.05) | | 0.274 | 0.93 (0.82, 1.05) | | 0.213 |
| Neu/Lym ^b^ | 1.19 (1.07, 1.33) | **0.002** | 1.17 (1.04, 1.31) | | **0.009** | 1.17 (1.05, 1.31) | | **0.006** | 1.14 (1.02, 1.28) | | **0.027** |
| PLT/Lym ^b^ | 1.01 (1.00, 1.01) | **0.000** | 1.01 (1.00, 1.01) | | **0.000** | 1.01 (1.00, 1.01) | | **0.000** | 1.01 (1.00, 1.01) | | **0.000** |
| IgG (g/L) | 1.06 (0.99, 1.13) | 0.105 | 1.06 (0.99, 1.15) | | 0.109 | 1.05 (0.98, 1.12) | | 0.202 | 1.05 (0.97, 1.13) | | 0.268 |
| IgM (g/L) | 0.72 (0.48, 1.07) | 0.108 | 0.81 (0.54, 1.22) | | 0.314 | 0.88 (0.58, 1.35) | | 0.563 | 0.98 (0.64, 1.51) | | 0.928 |
| CRP (mg/L) ^b^ | 1.02 (1.01, 1.03) | **0.000** | 1.02 (1.01, 1.03) | | **0.000** | 1.02 (1.01, 1.02) | | **0.000** | 1.02 (1.01, 1.02) | | **0.001** |
| ESR (mm/h) ^b^ | 1.02 (1.01, 1.03) | **0.000** | 1.02 (1.01, 1.03) | | **0.000** | 1.02 (1.01, 1.03) | | **0.000** | 1.02 (1.01, 1.03) | | **0.000** |
| **Glucose** |  |  |  | |  |  | |  |  | |  |
| GLU (mmol/L) | 1.24 (0.73, 2.12) | 0.430 | 1.79 (0.97, 3.30) | | 0.064 | 1.34 (0.78, 2.32) | | 0.290 | 1.99 (1.06, 3.75) | | **0.033** |

**Table notes:** ORs are calculated from binary logistic analysis. Patients were divided into two groups according to low (1+ and 2+) and high (3+ and 4+) sputum bacteria counts.

BMI, body mass index; History of TB, time elapsed between onset of symptoms and admittance to SPH. 0=No symptoms; RBC=red blood cells; HGB=haemoglobin; MCH=mean corpuscular haemoglobin; MCHC=mean corpuscular haemoglobin concentration; MCV= mean corpuscular volume; RDW-CV=red blood cell distribution width-coefficient of variation; RDW-SD=red blood cell distribution width standard deviation; P-LCR=platelet large cell ratios; PLT=counts of platelet; APTT=activated partial thromboplastin time; AT3=antithrombin III ratios; TT=thrombin time; FDP =fibrin degradation product; PCT=plateletcrit; HRCT=high-resolution computed tomography scores of the lungs; Neu=neutrophil ratios; BASO=basophil; EO=eosinophilia; Mon=monocyte; Neu=neutrophil; WBC=white blood cells; Neu/Lym=neutrophil to lymphocyte ratio; PLT/Lym=platelet to lymphocyte ratio; IgG= immunoglobulin G; IgM= immunoglobulin M; CRP=C-reactive protein; ESR=erythrocyte sedimentation rate; GLU=fasting glucose. See supplementary Table 3 and 4 legends for expansion of other abbreviation.

^a^ negative group as reference. ^b^ indice with *P*<0.05, but the value of aOR ratio (95% CI) includes /close to 1.000.

**Table S6 Association of immune, biochemical indices with cavity in 228 TB patients.**

| **Parameters** | **Unadjusted** | | | **Adjusted for age, BMI** | | | **Adjusted for sex** | | **Adjusted for sex, age, BMI** | | | | | |
| --- | --- | --- | --- | --- | --- | --- | --- | --- | --- | --- | --- | --- | --- | --- |
|  | **OR (95% CI)** | | ***P*** | **OR (95% CI)** | ***P*** | **OR (95% CI)** | | ***P*** | **OR (95% CI)** | | ***P*** | | |  |
|  |  |  |  |  |  |  |  |  |  |  |  |  |  |  |
| **Demographic indices** | | | | | | | | | | | | | |  |
| Age (year) | 0.99 (0.97, 1.01) | 0.442 | | - | - | 0.99 (0.97, 1.01) | | 0.481 | - | | - | |  |  |
| Weight (kg) ^b^ | 1.01 (0.97, 1.04) | 0.652 | | 1.10 (1.04, 1.17) | **0.002** | 0.95 (0.90, 0.99) | | **0.018** | 0.96 (0.87, 1.06) | | 0.400 | |  |  |
| Height (cm) ^b^ | 1.07 (1.03, 1.11) | **0.001** | | 1.07 (1.03, 1.12) | **0.001** | 1.00 (0.94, 1.06) | | 0.968 | 0.99 (0.93, 1.06) | | 0.813 | |  |  |
| BMI ^b^ | 0.90 (0.80, 1.02) | 0.090 | | - | - | 0.85 (0.74, 0.98) | | **0.023** | - | | - | |  |  |
| TB-Ab ^a^ | 0.71 (0.40, 1.26) | 0.239 | | 0.83 (0.46, 1.51) | 0.540 | 0.72 (0.39, 1.32) | | 0.284 | 0.83 (0.44, 1.58) | | 0.578 | |  |  |
| T-spot ^a^ | 1.01 (1.00, 1.02) | 0.293 | | 1.01 (1.00, 1.02) | 0.252 | 1.01 (1.00, 1.02) | | 0.352 | 1.01 (0.99, 1.02) | | 0.342 | |  |  |
| **Red blood cell-associated indices** | | | | |  |  | |  |  | |  | |  |  |
| RBC (×10^9^/L) | 0.62 (0.36, 1.05) | 0.076 | | 0.63 (0.35, 1.15) | 0.130 | 0.22 (0.11, 0.48) | | **0.000** | 0.21 (0.09, 0.48) | **0.000** | |  |  |  |
| HCT (%) | 0.00 (0.00, 1.67) | 0.072 | | 0.01 (0.00, 10.72) | 0.193 | 0.00 (0.00, 0.00) | | **0.000** | 0.00 (0.00, 0.00) | **0.000** | |  |  |  |
| HGB (g/L) ^b^ | 0.98 (0.97, 1.00) | **0.041** | | 0.99 (0.97, 1.00) | 0.121 | 0.95 (0.93, 0.97) | | **0.000** | 0.95 (0.93, 0.98) | **0.000** | |  |  |  |
| MCH (pg) | 0.96 (0.84, 1.09) | 0.487 | | 0.99 (0.87, 1.13) | 0.872 | 0.93 (0.81, 1.07) | | 0.335 | 0.99 (0.85, 1.14) | 0.832 | |  |  |  |
| MCHC (g/L) | 0.97 (0.95, 1.00) | 0.052 | | 0.97 (0.95, 1.00) | 0.091 | 0.96 (0.93, 0.99) | | 0.004 | 0.96 (0.93, 0.99) | 0.022 | |  |  |  |
| MCV (fL) | 1.00 (0.96, 1.05) | 0.905 | | 1.02 (0.97, 1.07) | 0.557 | 1.01 (0.96, 1.06) | | 0.807 | 1.02 (0.97, 1.08) | 0.423 | |  |  |  |
| RDW-CV (%) ^b^ | 1.28 (1.01, 1.62) | **0.041** | | 1.26 (0.99, 1.62) | 0.066 | 1.27 (0.99, 1.64) | | 0.062 | 1.22 (0.94, 1.58) | 0.144 | |  |  |  |
| RDW-SD (fL) ^b^ | 1.11 (1.02, 1.21) | **0.022** | | 1.12 (1.02, 1.24) | **0.017** | 1.11 (1.02, 1.22) | | **0.023** | 1.12 (1.02, 1.24) | **0.022** | |  |  |  |
| **Platelet and coagulation indices** | | | | |  |  | |  |  | |  | |  |  |
| P-LCR (%) ^b^ | 0.94 (0.91, 0.98) | **0.004** | | 0.95 (0.91, 0.99) | **0.007** | 0.95 (0.91, 0.99) | | **0.011** | 0.95 (0.92, 1.00) | | **0.026** | |  |  |
| PLT(×10^9^/L) | 1.00 (1.00, 1.01) | 0.388 | | 1.00 (1.00, 1.00) | 0.765 | 1.00 (1.00, 1.00) | | 0.522 | 1.00 (1.00, 1.00) | | 0.985 | |  |  |
| PCT (%) | 0.92 (0.03, 28.27) | 0.962 | | 0.43 (0.01, 15.45) | 0.643 | 1.12 (0.03, 44.00) | | 0.950 | 0.37 (0.01, 18.41) | | 0.620 | |  |  |
| PDW (%) | 0.94 (0.85, 1.03) | 0.170 | | 0.82 (0.68, 0.98) | 0.029 | 0.95 (0.86, 1.05) | | 0.294 | 0.83 (0.69, 1.01) | | 0.056 | |  |  |
| APTT (s) ^b^ | 1.09 (1.02, 1.15) | **0.010** | | 1.08 (1.01, 1.15) | **0.023** | 1.08 (1.01, 1.15) | | **0.027** | 1.07 (1.00, 1.15) | | 0.059 | |  |  |
| AT3 (%) ^b^ | 0.98 (0.96, 1.00) | **0.029** | | 0.98 (0.96, 1.00) | 0.085 | 0.99 (0.97, 1.01) | | 0.184 | 0.99 (0.97, 1.01) | | 0.410 | |  |  |
| TT (s) | 0.95 (0.84, 1.09) | 0.464 | | 0.94 (0.82, 1.08) | 0.369 | 0.97 (0.85, 1.12) | | 0.697 | 0.95 (0.82, 1.10) | | 0.527 | |  |  |
| D-Dimer(ng/mL) ^b^ | 1.00 (1.00, 1.00) | **0.007** | | 1.00 (1.00, 1.00) | **0.012** | 1.00 (1.00, 1.00) | | **0.015** | 1.00 (1.00, 1.00) | | **0.036** | |  |  |
| FDP (µg/mL) ^b^ | 1.13 (1.03, 1.24) | **0.011** | | 1.11 (1.02, 1.22) | **0.022** | 1.12 (1.02, 1.22) | | **0.021** | 1.09 (1.00, 1.19) | | 0.056 | |  |  |
| C3 (g/L) | 1.50 (0.55, 4.11) | 0.427 | | 1.61 (0.55, 4.71) | 0.385 | 1.56 (0.53, 4.55) | | 0.417 | 1.55 (0.49, 4.89) | | 0.450 | |  |  |
| **Immune cells and inflammatory indices** | | | | | |  | |  |  | |  | |  |  |
| BASO (%) | 0.75 (0.27, 2.07) | 0.581 | | 0.62 (0.20, 1.89) | 0.396 | 0.80 (0.28, 2.33) | | 0.683 | 0.71 (0.22, 2.29) | | 0.561 | |  |  |
| EO (%) | 0.97 (0.83, 1.13) | 0.664 | | 0.97 (0.82, 1.14) | 0.705 | 0.97 (0.82, 1.14) | | 0.685 | 0.98 (0.82, 1.18) | | 0.832 | |  |  |
| Lym (%) ^b^ | 0.95 (0.92, 0.98) | **0.002** | | 0.95 (0.92, 0.99) | **0.007** | 0.95 (0.92, 0.99) | | **0.011** | 0.96 (0.93, 1.00) | | **0.048** | |  |  |
| Mon (%) | 1.06 (0.95, 1.18) | 0.323 | | 1.05 (0.94, 1.18) | 0.361 | 1.03 (0.92, 1.15) | | 0.652 | 1.02 (0.91, 1.15) | | 0.701 | |  |  |
| Neu (%) ^b^ | 1.04 (1.01, 1.07) | **0.013** | | 1.03 (1.00, 1.07) | **0.031** | 1.03 (1.00, 1.07) | | **0.037** | 1.03 (1.00, 1.06) | | 0.104 | |  |  |
| BASO(×10^9^/L) | 0.00 (0.00, 9827.00) | 0.461 | | 0.00 (0.00, 282.60) | 0.197 | 0.00 (0.00, 12508.00) | | 0.437 | 0.00 (0.00, 1098.00) | | 0.242 | |  |  |
| EO(×10^9^/ L) | 1.47 (0.17, 12.87) | 0.728 | | 1.07 (0.09, 12.82) | 0.955 | 1.19 (0.12, 11.80) | | 0.884 | 0.99 (0.07, 14.61) | | 0.992 | |  |  |
| Mon(×10^9^/L) | 2.53 (0.75, 8.52) | 0.134 | | 2.14 (0.62, 7.40) | 0.230 | 1.23 (0.34, 4.42) | | 0.751 | 1.02 (0.27, 3.88) | | 0.976 | |  |  |
| Neu(×10^9^/L) | 1.07 (0.95, 1.20) | 0.279 | | 1.04 (0.93, 1.17) | 0.466 | 1.03 (0.91, 1.17) | | 0.624 | 1.01 (0.89, 1.13) | | 0.910 | |  |  |
| WBC(×10^9^/L) | 1.03 (0.93, 1.15) | 0.522 | | 1.02 (0.92, 1.12) | 0.760 | 1.00 (0.90, 1.11) | | 0.938 | 0.98 (0.88, 1.09) | | 0.703 | |  |  |
| Neu/Lym ^b^ | 1.18 (1.02, 1.36) | **0.022** | | 1.15 (1.00, 1.33) | 0.051 | 1.15 (1.00, 1.32) | | 0.052 | 1.11 (0.96, 1.29) | | 0.146 | |  |  |
| PLT/Lym ^b^ | 1.01 (1.00, 1.01) | **0.002** | | 1.01 (1.00, 1.01) | **0.005** | 1.01 (1.00, 1.01) | | **0.004** | 1.01 (1.00, 1.01) | | **0.015** | |  |  |
| IgG (g/L) | 1.04 (0.97, 1.12) | 0.289 | | 1.04 (0.96, 1.13) | 0.314 | 1.00 (0.93, 1.09) | | 0.915 | 0.99 (0.91, 1.08) | | 0.785 | |  |  |
| IgM (g/L) | 0.87 (0.59, 1.30) | 0.507 | | 0.90 (0.59, 1.36) | 0.606 | 1.20 (0.77, 1.86) | | 0.420 | 1.22 (0.77, 1.93) | | 0.406 | |  |  |
| CRP (mg/L) ^b^ | 1.03 (1.02, 1.04) | **0.000** | | 1.03 (1.01, 1.04) | **0.000** | 1.02 (1.01, 1.04) | | **0.000** | 1.02 (1.01, 1.03) | | **0.001** | |  |  |
| ESR (mm/h) ^b^ | 1.02 (1.01, 1.03) | **0.000** | | 1.02 (1.01, 1.03) | **0.001** | 1.02 (1.01, 1.03) | | **0.000** | 1.02 (1.01, 1.03) | | **0.002** | |  |  |
| **Glucose** |  |  | |  |  |  | |  |  | |  | |  |  |
| GLU (mmol/L) | 0.87 (0.49, 1.54) | 0.631 | | 1.16 (0.61, 2.19) | 0.650 | 0.98 (0.53, 1.80) | | 0.940 | 1.33 (0.67, 2.64) | | 0.416 | |  |  |

**Table notes:** ORs are calculated from binary logistic analysis. See supplementary Table 3,4 and 5 legends for expansion of other abbreviation.

^a^ negative group as reference. ^b^ index with *P*<0.05, but the value of OR ratios (95% CI) includes /close to 1.000.
