# Supplementary figures and images for: Sex influences the association between haemostasis and the extent of lung lesions in tuberculosis
Source: Biol Sex Differ. 2018 Oct 10;9:44. doi: 10.1186/s13293-018-0203-9 (PMC6180492; doi:10.1186/s13293-018-0203-9)

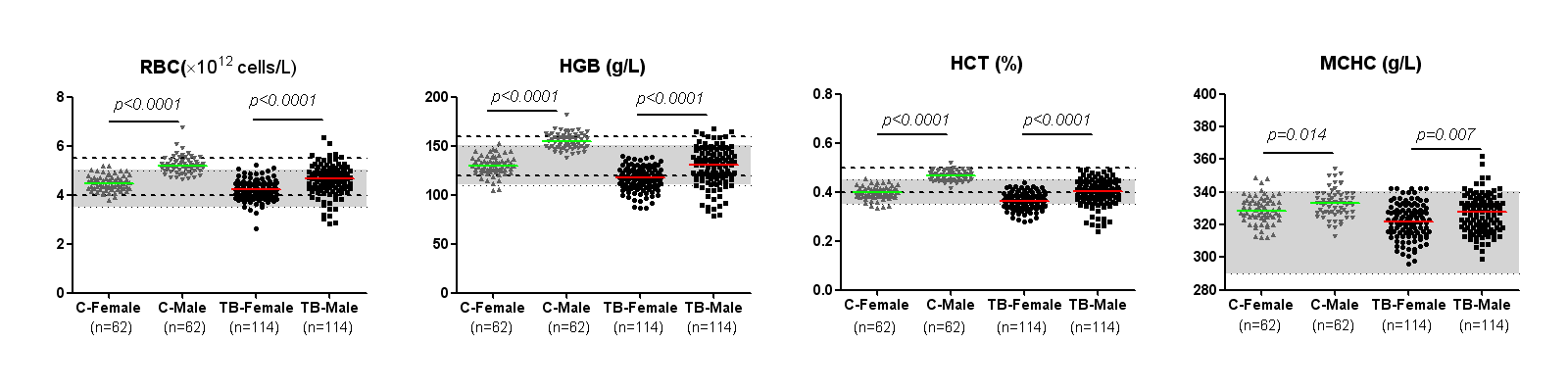

Supplement: Supplementary file 2 — Figure S1. Levels of red blood cell-associated indices in male and female healthy control and TB patients. Horizontal lines represent median values. Grey areas represent the normal ranges of the index in women; the area between dashed lines represent the normal ranges of the index in men. The differences between groups were analysed by Mann-Whitney U tests. RBC, red blood cell; HGB, haemoglobin; HCT, haematocrit; MCHC, mean corpuscular hemoglobin concentration. (TIF 2118 kb) [file 13293_2018_203_MOESM2_ESM.tif]

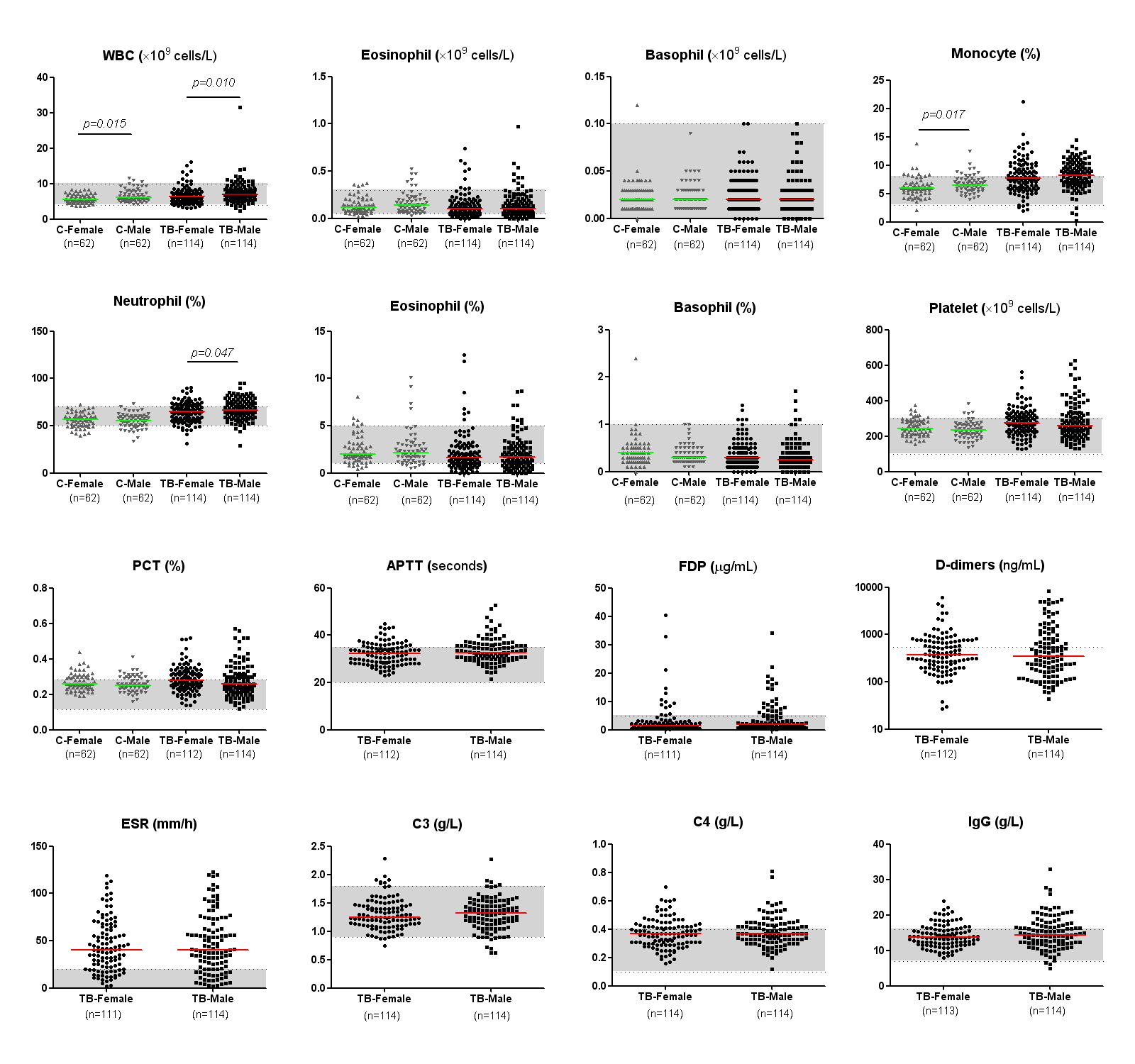

Supplement: Supplementary file 3 — Figure S2. The indices with no or negligible differences between male and female TB patients. Horizontal lines represent median values. Grey areas represent the normal ranges of the indices. The differences between groups were analysed by Mann-Whitney U tests. WBC, white blood cell; PCT, plateletcrit; APTT, activated partial thromboplastin time; C3, complement 3; C4, complement 4; FDP, fibrinogen degradation product; IgG, immunoglobulin G. (TIF 7818 kb) [file 13293_2018_203_MOESM3_ESM.tif]
